# Abnormal glucose regulation in Chinese patients with coronary artery disease: A cross-sectional study: Erratum

**DOI:** 10.1097/MD.0000000000010024

**Published:** 2018-02-23

In the article, “Abnormal glucose regulation in Chinese patients with coronary artery disease: A cross-sectional study”,^[1]^ which appeared in Volume 96, Issue 52 of *Medicine*, the authors need to add the following funding information "This study is supported by the Project (No. 2006BAI01A02) supported by the 11th 5-Year National Key Technologies R&D Program for Coronary Heart Disease (CHD) from the Ministry of Science and Technology of China.”
